# A brief xerostomia inventory for young adults

**DOI:** 10.1371/journal.pone.0349869

**Published:** 2026-05-20

**Authors:** Benjamin W. Chaffee, Elizabeth T. Couch, Caroline Shiboski, Lindsay Till Hoyt, Alison K. Cohen, Jing Cheng

**Affiliations:** 1 Division of Oral Epidemiology and Dental Public Health, University of California San Francisco, San Francisco, California, United States of America; 2 Philip R. Lee Institute for Health Policy Studies, University of California San Francisco, San Francisco, California, United States of America; 3 Department of Orofacial Sciences, University of California San Francisco, San Francisco, California, United States of America; 4 Department of Psychology, Fordham University, New York, New York, United States of America; 5 Department of Epidemiology and Biostatistics, University of California San Francisco, San Francisco, California, United States of America; PGIMER: Post Graduate Institute of Medical Education and Research, INDIA

## Abstract

**Objective:**

Xerostomia (dry mouth) is a common condition that negatively impacts life quality for adults of all ages. Yet, xerostomia has primarily been studied among older adults and populations at high risk of dry mouth, with no widely-available self-reported instrument to assess xerostomia accurately among younger populations. This study aimed to develop a brief xerostomia assessment tool specifically designed for young adults.

**Methods:**

Students ages 18–24 at two California (USA) public universities (N = 278) completed a cross-sectional survey that included a 14-item xerostomia inventory, including new items and items previously tested among older adults. Using exploratory factor analysis and item response theory analysis, the inventory was reduced to a 5-item scale. Psychometric properties of the 5 items were examined. As further validity checks, associations were explored between scale sum scores and other oral health conditions and potential xerostomia risk indicators.

**Results:**

A proposed 5-item Young Adult version of the Shortened Xerostomia Inventory (SXI-Y) had favorable psychometric properties in this population, including excellent internal consistency (Cronbach alpha = 0.817) and strong correlation with a global measure of dry mouth (polychoric correlation = 0.711). SXI-Y sum scores (overall mean = 7.3; median = 6; range: 5–18) were higher (indicating more severe xerostomia) among participants who reported gum bleeding, oral pain, and halitosis and those taking prescription medications, using nicotine or cannabis, and reporting less sleep and more stress.

**Conclusions:**

In this study, a xerostomia measure tailored for young adults demonstrated stronger internal consistency and retained more item-level information than an earlier tool developed for older adults. Associations of xerostomia with sleep and stress merit further investigation, as does instrument testing in additional study populations.

## Introduction

Xerostomia, the subjective experience of dry mouth, is a common and uncomfortable condition that may compromise daily functions, such as eating and speaking, and elevate risk for other adverse oral conditions, including dental caries lesions and oral fungal infections [1–3]. Xerostomia is most well studied among older adults (typically, ages 60 or above), for whom a higher burden of prescription drugs, especially medications with anticholinergic side-effects, can dramatically increase dry mouth experience [4,5]. However, growing evidence suggests that xerostomia can be common across the life course, including in young adulthood [6–8]. Investigating xerostomia across a wider age range could advance the evidence base on emerging correlates, such as nicotine and cannabis use, stress, and sleep quality, that could inform xerostomia management for affected patients. However, existing instruments to measure xerostomia have not been validated for use among younger adults.

Valid instruments to measure dry mouth experience are essential to study xerostomia clinically and in community populations. However, the most widely used xerostomia survey instruments were developed and tested near-exclusively among older adults, while few, if any, instruments have been rigorously tested among younger populations. For example, the Xerostomia Inventory (XI) was developed as an 11-item summed scale among adults aged 65 and older [9]. Later, the XI was reduced to a 5-item Shortened Xerostomia Inventory (SXI, also called the “Summated Xerostomia Inventory” or “Summated Xerostomia Inventory–Dutch Version”) and validated among six populations of older adults [10,11].

The SXI has been used to demonstrate associations between xerostomia and several other conditions, including radiotherapy for head and neck cancer [12], cannabis use [13], and symptoms of dry eye [14]. The SXI has also been translated and tested in numerous languages, such as Chinese [15], Thai [16], German [17], and Korean [18], among others. However, it is possible that the SXI might not perform adequately among younger populations, who might experience dry mouth differently, even if the etiologies of the condition are similar. For example, two SXI items relate to swallowing and eating difficulties, which may manifest only under severe dry mouth. Additionally, an SXI item related to dry lips, a highly prevalent condition, may not be sufficiently specific to xerostomia. Developing a xerostomia instrument with sufficient sensitivity and specific for use with younger adults could permit more extensive study of dry mouth among non-elder populations.

Therefore, the goal of the present study was to develop and test a brief xerostomia inventory for use with young adult populations. This goal was achieved by implementing a survey-based cross-sectional study of xerostomia among university students that was built on the infrastructure of the Economic and Educational Contributors to Emerging Adults’ Cardiometabolic and Oral Health (3E) Study.

## Methods

### Study design and population

The 3E Study is an ongoing investigation of economic and education-related influences of cardiometabolic and oral health among young adult undergraduate university students enrolled in two public universities in California (USA). Full-time undergraduate students aged 18–24 can join the 3E cohort in their first or second year (or first transfer-student year). The present cross-sectional analysis examines the results of an ancillary survey that (in addition to investigating xerostomia) aimed to enhance engagement and enrollment in the main 3E Study. From January 24 to June 2, 2025, currently enrolled 3E Study participants and members of the 3E Youth Advisory Board (who are also undergraduate students at these universities) were sent email and text invitations to participate in the ancillary dry mouth survey. Additionally, any other full-time first- or second-year or newly transferred students at either participating university could take part and were reached via fliers, social media posts, and paid campus newspaper advertisements. There were no exclusions for diabetes or other systemic conditions that could affect xerostomia or other aspects of oral health, given their low prevalence among younger populations. At the conclusion of the dry mouth survey (median completion time: 5 minutes), participants could enroll themselves in the main 3E Study (if not already enrolled) or refer another student to be invited to the survey.

### Ethics statement

Before beginning the survey, participants viewed an online informed consent statement describing the study and indicated their willingness to participate by clicking an electronic link to continue. Given the low-risk nature of the study, the Institutional Review Board (IRB) did not require a written signature. Participants received a $5 electronic gift card for completing the dry mouth survey. The Fordham University IRB approved all study procedures as the single IRB on which all collaborating sites relied (Protocol #2264).

### Xerostomia measures

A single global xerostomia item asked participants, “How often does your mouth feel dry?” (options: never, occasionally, frequently, always) [19]. To construct a numeric sum scale of dry mouth experience, 14 individual xerostomia items were presented in an individually randomized order within a list (options: never, rarely, sometimes, often, very often), similarly to earlier XI and SXI development (Table 1) [9–11]. Nine of the 14 items were part of the original XI, including 5 that were retained in the SXI. Five other items were worded specifically for this analysis based on discussion with co-authors and Youth Advisory Board members, although some featured concepts, such as taste and speech disturbances, had been raised in prior literature [9,20,21].

**Table 1:** Participant Responses to Xerostomia Scale Items (N = 278).

	Responses, %				
Item	Never	Rarely	Some-times	Often	Very Often
1. I sip liquid to help swallow my food^a^	55.4	20.1	16.9	5.8	1.8
2. My mouth feels dry when eating a meal^a^*	68.7	23.0	7.6	0.7	0
3. I have difficulty eating dry foods^b^*	71.6	18.0	8.6	1.4	0.4
4. I use mints, candy, or cough drop to relieve dry mouth^c^	69.8	15.5	11.9	2.5	0.4
5. I have difficulty swallowing certain foods^a^*	76.6	16.9	5.8	0.7	0
6. My eyes feel dry^b^	42.1	23.7	20.5	10.8	2.9
7. My lips feel dry^b^*	13.7	19.8	41.7	16.2	8.6
8. My mouth feels dry^d^*	40.3	37.4	16.5	4.0	1.8
9. I sip liquids all day to keep my mouth from drying out^e^	47.8	19.4	16.9	11.2	4.7
10. I feel like I don’t have enough saliva^f^	63.7	23.4	11.2	1.8	0
11. It feels like there are cotton balls in my mouth^e^	81.7	14.0	4.0	0.4	0
12. Food tastes funny because my mouth is dry^e^	83.5	9.7	6.5	0.4	0
13. It’s hard to talk because my mouth is dry^e^	75.2	17.3	6.8	0.7	0
14. My mouth is too dry to whistle^e^	75.9	15.5	6.5	1.1	1.1

a. Sources: Thomson, et al. 1999; Fox, et al. 1987

b. Source: Thomson, et al. 1999

c. Adapted from: Thomson, et al. 1999

d. Sources: Thomson, et al. 1999; Thomson, et al. 1993

e. Newly worded item

f. Adapted from: Fox, et al. 1987

*Items included in the Shortened Xerostomia Inventory (van der Putten, et al. 2011; Thomson, et al. 2011)

Notes: Participants were asked, “How often did you experience the following in the PAST 30 DAYS?” All 14 items were presented in individually randomized order within a list. Response options to each item were: Never, Rarely, Sometimes, Often, Very often.

### Other oral health variables

Participants were asked to “rate the health of your teeth and gums” from excellent to poor [22]. In separate questions, participants were asked whether in the past six months they experienced “any bleeding after brushing or flossing, or due to other conditions in your mouth,” how often “have you had pain anywhere in your mouth?” (dichotomized for analysis as 1 = sometimes, often, very often; 0 = never, rarely), and “have you had a cavity (tooth decay) in any of your teeth?” [23] They were also asked, “Do you think you have halitosis (bad breath)?” [24].

### Potential dry mouth correlates

Participants reported their age (years), gender (male, female, something else), and overall health (excellent, very good, good, fair, poor). Participants self-reported their height (feet and inches) and weight (pounds), which were converted to body mass index. Participants were asked if they are “now taking any medication prescribed by a doctor” and, if yes, how many. In separate questions, participants were asked, “During the past 30 days, on how many days” did they use cigarettes or other combustible tobacco, cannabis, e-cigarettes (“vapes”), or nicotine pouches. Substance use was defined as use of any of these products on at least one day. Participants reported their hours of sleep on a “typical night” and rated their sleep quality from very good to very poor [25]. Responses to the 10-item Perceived Stress Scale [26] were combined to sum score and, given a normal distribution, categorized as beyond or within 1 standard deviation below or above the sample mean.

### Psychometric analyses

A total of N = 285 non-duplicate, eligible surveys were completed. Analysis was restricted to N = 278 participants with no missing data on any of the 14 individual xerostomia scale items. Following previous xerostomia scale development [9–11], exploratory factor analysis (principal factors method unrotated) was conducted to describe correlation of individual item responses to any hypothetical latent dry mouth construct and assess the dimensionality of the questionnaire. A unidimensional instrument is considered to measure one underlying factor or construct (here, dry mouth). Additionally, item response theory (IRT) analysis using a graded response model for ordinal variables (an extension of the 2-parameter logistic IRT) was completed to assess how items measure the latent dry mouth construct and how participants differ in their responses A set of difficulty (threshold) parameters and a discrimination (slope) parameter were estimated for each scale item. A difficulty coefficient mapped xerostomia scale items along a latent variable (theta), separating items from “easiest” to “hardest” endorse, loosely approximating less to more severe xerostomia experience. Consistently increasing threshold parameters indicate that participants with more severe xerostomia are more likely to choose higher response categories. A discrimination coefficient estimated how well each item distinguished between participants below or above a given level of xerostomia (theta). Prior work has described item discrimination as follows: very low (exceeding 0.00), low (0.35), moderate (0.65), high (1.35), and very high (1.70) [27].

To select items for a potential young adult version of the Shortened Xerostomia Inventory (SXI-Y), co-authors examined factor loadings and IRT coefficients with both statistical and clinical considerations. Included items were required to be in the upper median of factor loadings. Preferred were individual items with higher IRT discrimination coefficients and higher IRT item information curves whose threshold parameters cover the range of latent dry mouth, greater internal consistency and a higher IRT test information curve across the continuum of latent dry mouth when combined, and, when summed (see below), greater polychoric correlation with the global xerostomia item and greater area under a receiver operating characteristic curve.

The internal consistency and reliability of the 5-item SXI-Y was measured using Cronbach’s alpha. Higher summed scores on the SXI-Y indicated more severe xerostomia (minimum possible score: 5; maximum possible score: 25). Criterion validity of the SXI-Y was examined between the SXI-Y summed scores and the widely accepted global xerostomia item by polychoric correlation and Jonckheere-Terpstra test. Exploratorily, receiver operating characteristic curves and areas under the curve were estimated for the SXI-Y and original SXI using the global xerostomia item as a gold standard.

### Associations with other variables

Construct validity of the SXI-Y was assessed via predefined associations between putative dry mouth correlates and SXI-Y, and between SXI-Y and other oral health conditions [28]. Pairwise associations between SXI-Y sum scores and oral health variables or potential dry mouth risk factors were tested using Wilcoxon rank-sum and Kruskal-Wallis tests to account for a right-skewed score distribution (*P* < 0.05 considered statistically significant). Missing values on other variables were not imputed. Given the sample size, relatively homogenous population age and setting, and exploratory nature of the analysis, there was no adjustment for multiple comparisons or confounding variables. All analyses were conducted using Stata version 16.1.

## Results

Participants who completed all 14 xerostomia scale items (N = 278) ranged in age from 18 to 24 years, 67% identified as female, 44% identified as Asian ancestry and 39% as Hispanic/Latine, 9% described their overall health as excellent and 32% as very good. In this population, the overall frequency of experiencing dry mouth according to the single global xerostomia item (“How often does your mouth feel dry?”) was 33% never, 57% occasionally, 8% frequently, and 1% always. Of the 14 individual items (Table 1), those endorsed most often (i.e., prevalence of responding at least “sometimes”) were lips feel dry (67%), eyes feel dry (34%), and sip liquids all day (33%). The items endorsed least often were cotton balls in mouth (4%), difficulty swallowing certain foods (6%), and food tastes funny (7%).

In exploratory factor analysis, one factor had an eigenvalue of 4.97 and explained 91.8% of the variance. No other factor had an eigenvalue greater than 0.5, suggesting that a unidimensional model is reasonable for the data (S1 Fig). Table 2 shows the factor loadings on the largest factor for the 14 individual xerostomia scale items. In IRT analysis (Table 2; S2 Fig), items ranged in difficulty (theta) lips feel dry (lowest theta; least severe xerostomia) to food tastes funny (highest theta; most severe xerostomia) and in discrimination from 0.95 (lips feel dry) to 2.93 (not enough saliva).

**Table 2:** Factor loadings and item response coefficients.

		Item Response Analysis		
Item	Factor Loading^a^	Difficulty^b^ (rank)	Discrimination (coefficient)	Select for SXI-Y
10. I feel like I don’t have enough saliva	0.750	9	2.925	yes
8. My mouth feels dry*	0.704	12	2.193	yes
13. It’s hard to talk because my mouth is dry	0.675	5	2.418	yes
14. My mouth is too dry to whistle	0.669	4	2.342	yes
2. My mouth feels dry when eating a meal*	0.667	8	2.403	
11. It feels like there are cotton balls in my mouth	0.637	2	2.457	yes
3. I have difficulty eating dry foods*	0.636	7	2.212	
5. I have difficulty swallowing certain foods*	0.590	3	2.011	
12. Food tastes funny because my mouth is dry	0.590	1	2.386	
4. I use mints, candy, or cough drop to relieve dry mouth	0.493	6	1.556	
1. I sip liquid to help swallow my food	0.489	10	1.259	
9. I sip liquids all day to keep my mouth from drying out	0.471	11	1.309	
6. My eyes feel dry	0.444	13	1.041	
7. My lips feel dry*	0.399	14	0.953	

a. From exploratory factor analysis estimated by the principal factors method (unrotated)

b. Items ranked from most [1] to least [14] difficult, where greater difficulty (higher theta) approximates more severe xerostomia

*Items included in the Shortened Xerostomia Inventory (van der Putten, et al. 2011; Thomson, et al. 2011)

Abbreviation: SXI-Y = Young Adult Shortened Xerostomia Inventory

Five items were selected for the SXI-Y. One of the SXI-Y items (mouth feels dry) was part of the original SXI (Table 2). The five items selected for the SXI-Y were internally consistent (Cronbach alpha = 0.817). This was better internal consistency than reached in this dataset with the five items comprising the original SXI (Cronbach alpha = 0.715). Compared to the original SXI, the SXI-Y items maintained more of the IRT information provided by all 14 xerostomia scale items (S3 Fig).

SXI-Y sum scores were strongly correlated with the single global xerostomia item (polychoric rho: 0.711; Jonckheere-Terpstra *P* < 0.001) (S4 Fig). Based on receiver operating characteristic curves, the SXI-Y had a slightly higher but not statistically significantly different area under the curve than the original SXI for occurrences of dry mouth frequently or always (SXI-Y: 0.885; SXI: 0.875; *P* = 0.699). However, the SXI-Y had a statistically significantly higher area under the curve for occurrences of dry mouth occasionally, frequently, or always (SXI-Y: 0.887; SXI: 0.810; *P* = 0.002) (S5 Fig).

SXI-Y sum scores were correlated with self-reported oral health status (Table 3). Scores were higher (indicating more frequent dry mouth-related experiences) among participants who reported fair or poor oral health, gum bleeding, oral pain, or bad breath (Table 3). SXI-Y sum scores were also correlated with potential dry mouth risk factors, including fair/poor overall health, medication use, substance use (tobacco, nicotine, or cannabis), shorter sleep duration, and greater perceived stress (Table 4).

**Table 3:** Associations between Oral Conditions and Young Adult Shortened Xerostomia Inventory Score.

		SXI-Y Score		
Oral Condition	N	median	mean	p-value^a^
Self-Rated Oral Health				
Excellent	21	6	7.1	0.03
Very Good	72	6	6.8	
Good	112	6	7.1	
Fair	63	7	8.1	
Poor	8	9	9.3	
Gum Bleeding				
Yes	133	7	7.8	<0.001
No	134	6	6.8	
Oral Pain				
Yes	74	7	8.1	<0.001
No	200	6	7.0	
Dental Cavity				
Yes	52	7	7.8	0.23
No	188	6	7.1	
Bad Breath				
Yes	39	8	8.9	<0.001
No	169	6	6.6	

a. Two-sample Wilcoxon rank-sum test for two-category variables; Jonckheere-Terpstra test for ordinal category variables (i.e., self-rated oral health)

Abbreviation: SXI-Y = Young Adult Shortened Xerostomia Inventory

**Table 4:** Associations between Potential Dry Mouth Correlates and Young Adult Shortened Xerostomia Inventory Score.

		SXI-Y Score		
Participant Variable	N	median	mean	p-value^a^
Self-Rated Overall Health				
Excellent	26	6	6.7	<0.001
Very Good	88	6	6.7	
Good	113	6	7.2	
Fair/Poor	51	8	9.1	
Age				
18 years	71	6	7.5	0.24
19 years	110	6	7.4	
20 years	48	6	6.9	
≥21 years	45	6	7.4	
Gender				
Female	186	6	7.5	0.33
Male	81	6	6.9	
Other response	10	8.5	8.9	
Body Mass Index				
<18.5	24	7	7.9	0.56
18.5 to 24.99	154	6	7.3	
25 to 29.99	43	6	6.6	
≥30	31	7	7.9	
Number of Medications				
None	221	6	7.1	0.002
One	30	8	8.5	
Two or more	21	7	8.2	
Substance Use^b^				
Yes	42	8	8.0	0.01
No	232	6	7.2	
Sleep Duration				
3 to 5.5 hours	23	8	8.9	0.03
6 to 7.5 hours	168	6	7.2	
≥8 hours	83	6	7.0	
Sleep Quality				
Very Good	22	7	7.1	0.22
Good	119	6	6.9	
Fair	106	6	7.5	
Poor/Very Poor	27	7	8.0	
Perceived Stress				
>1 SD below mean	44	6	6.4	<0.001
< 1 SD below mean	93	6	6.7	
< 1 SD above mean	90	6.5	7.5	
> 1 SD above mean	48	8	8.9	

a Two-sample Wilcoxon rank-sum test for two-category variables (i.e., any substance use); Kruskal-Wallis test for ≥3-category nominal variables (i.e., gender); Jonckheere-Terpstra test for ordinal category variables (e.g., self-rated overall health)

b Use of cigarettes or other combustible tobacco, cannabis, e-cigarettes, and/or nicotine pouches at least once in the past 30 days

Abbreviations: SD = standard deviation; SXI-Y = Young Adult Shortened Xerostomia Inventory

## Discussion

In this study of young adult undergraduate university students, xerostomia was a somewhat common experience, with approximately 10% of the sample frequently or always experiencing dry mouth. This prevalence was similar but slightly higher than national estimates from the USA (ages 18–24: 7%) [8] and Australia (ages 15–34: 9%) [7]. Based on this university population, a 5-item instrument, the Young Adult Shortened Xerostomia Inventory (SXI-Y), was developed, tested, and demonstrated favorable psychometric properties. While the SXI-Y is similar to the existing Shortened Xerostomia Inventory (SXI), which was developed exclusively among older adults, the SXI-Y had moderately better internal consistency and receiver operating characteristics. SXI-Y sum scores were associated with established xerostomia risk factors, such as medication use, and with emerging correlates, like substance use, stress, and sleep, suggesting its utility for further studies of dry mouth in age-diverse populations.

Among study limitations, the cross-sectional design does not allow evaluation of xerostomia over time, including in relation to potential risk factors and health-related consequences. Also, results from a sample of young adult university students may not necessarily generalize to all young adults, despite covering a range of socioeconomic backgrounds. The initial findings from this one sample, both SXI-Y properties and associations with other variables, should be confirmed in additional populations. Advantageously, this study featured a triangulated approach to instrument development, relying on insight from multiple statistical methods (e.g., factor analysis, item response theory) to build the SXI-Y scale [29]. Thus, the SXI-Y should have both strong correlation with any hypothetical underlying dry mouth factor and ability to discriminate between populations with and without xerostomia over a range of dry mouth severity.

SXI-Y scores in the present study were associated with use of nicotine and/or cannabis; however, the number of participants using any particular tobacco, nicotine, or cannabis product was too small to examine each product separately. This finding is consistent with recent work from a national study of adults that found each of cigarette, e-cigarette, and cannabis use to be associated with xerostomia [8]. Additionally, greater perceived stress was also associated with xerostomia in the present sample. Similar relationships were reported in studies of dental students and patients visiting a saliva clinic [30,31]. It is possible that xerostomia may engender stress or that chronic stress may impact salivary function and/or dry mouth perception [32]; prospective studies are needed to evaluate this relationship further.

In the present study, according to SXI-Y score, xerostomia was more severe among participants who slept fewer hours per night and reported worse sleep quality (albeit not statistically significantly for sleep quality). This mirrors a prior study of male military service members in Greece, which reported associations of xerostomia with poor sleep quality and excessive daytime sleepiness [33]. Salivary flow follows a circadian pattern, with less salivation overnight [34]. Speculatively, overnight dry mouth symptoms that cause waking (for example, to drink water [33]) or disrupt breathing (for example, by increasing surface tension in the upper airway [35]) could negatively impact sleep quality. Confirming this association and further examining the direction of the sleep-xerostomia relationship merits additional study.

In this analysis, participants taking any prescription medication had higher SXI-Y scores than those taking none. We did not ask participants which medications they were prescribed. Many types of medications have been associated with xerostomia, particularly those with anticholinergic effects, such as antihistamines, antidepressants, antipsychotics, antianxiety medications, and decongestants [1,2]. Some of these medication classes are commonly used among young adults, however, the relatively small number of participants prescribed medications in this study sample did not permit an in-depth analysis by medication type. Similarly, given their low prevalence, systemic conditions, such as diabetes, were not examined in this investigation. Availability of a valid xerostomia instrument for young adults could permit larger future studies of xerostomia risk factors, including systemic diseases.

Treatment approaches for xerostomia largely aim to manage symptoms, such as encouraging hydration, use of chewing gum or lozenges to stimulate saliva flow, or over-the-counter saliva stimulants or substitutes [3]. Patients taking xerogenic medications may discuss with their healthcare team substitute medicines or dose reduction [3]. Prescribing daily topical fluoride rinses or gels, is one approach to reduce caries risk among patients with medication-induced dry mouth [1,2]. Particularly relevant for younger adults but virtually unexamined, are the long-term implications of xerostomia over the life course, including the physical and psychological impacts on quality-of-life and the most effective and sustainable management strategies. The SXI-Y proposed here offers of tool for such investigations.

Other questions needing further investigation include affirming SXI-Y validity in additional populations, such as those with lower literacy levels than university students. Also, prospective studies are required to examine how SXI-Y scores change over time, which would allow calculation of a minimally important difference in SXI-Y scores that aligns with meaningful improvement in dry mouth experience. How the psychometric performance of the SXI-Y compares to the SXI in populations of middle-aged adults is another question worthy of future study. A prior Rasch analysis of the SXI suggested similar instrument performance with fewer response options [36]; a similar analysis could be carried out for the SXI-Y. Future studies that incorporate additional clinical factors could also provide further insight about xerostomia etiology or the causes of potentially related conditions, such as halitosis. For example, the present study did not measure objective salivary flow rate, water intake, or clinical outcomes, like caries lesions. Such studies could relate these factors to the subjective experience of dry mouth, leveraging the SXI-Y to extend these investigations to younger populations.

In summary, the results of this study suggest that the SXI-Y has utility as a brief, valid xerostomia scale among young adults. Further testing should be conducted in additional populations, both to test and potentially refine the instrument and to confirm emerging associations with sleep and stress, which could be important elements in managing dry mouth and improving quality-of-life among xerostomia patients. The availability of xerostomia measurement tools that have been tested in age-diverse samples can expand research on this condition to wider populations.

## Supporting information

S1 File Supporting information. This Supporting Information files contains the additional figures S1 Fig, through S5 Fig. and their captions.
(PDF)

## References

[1] Guggenheimer J, Moore PA. Xerostomia: etiology, recognition and treatment. J Am Dent Assoc. 2003;134(1):61–9; quiz 118–9. doi: 10.14219/jada.archive.2003.0018 12555958

[2] Greenspan D. Xerostomia: diagnosis and management. Oncology (Williston Park). 1996;10(3 Suppl):7–11. 8723427

[3] Villa A, Connell CL, Abati S. Diagnosis and management of xerostomia and hyposalivation. Ther Clin Risk Manag. 2014;11:45–51. doi: 10.2147/TCRM.S76282 25653532 PMC4278738

[4] Thomson WM, Chalmers JM, Spencer AJ, Slade GD. Medication and dry mouth: findings from a cohort study of older people. J Public Health Dent. 2000;60(1):12–20. doi: 10.1111/j.1752-7325.2000.tb03286.x 10734611

[5] Stenbäck J, Tiisanoja A, Syrjälä A-M, Komulainen K, Hartikainen S, Ylöstalo P. High anticholinergic burden and hyposalivation and xerostomia in the elderly. Acta Odontol Scand. 2023;81(6):436–42. doi: 10.1080/00016357.2023.2166105 36628441

[6] Agostini BA, Cericato GO, da Silveira ER, Nascimento GG, Costa FDS, Thomson WM, et al. How common is dry mouth? Systematic review and meta-regression analysis of prevalence estimates. Braz Dent J. 2018;29(6):606–18. doi: 10.1590/0103-6440201802302 30517485

[7] Jamieson LM, Thomson WM. Xerostomia: its prevalence and associations in the adult Australian population. Aust Dent J. 2020;65 Suppl 1:S67–70. doi: 10.1111/adj.12767 32583587

[8] Chaffee BW. Associations of current cigarette, E-cigarette, and Cannabis use with Xerostomia. JDR Clin Trans Res. 2025;:23800844251364158. doi: 10.1177/23800844251364158 40908802

[9] Thomson WM, Chalmers JM, Spencer AJ, Williams SM. The Xerostomia Inventory: a multi-item approach to measuring dry mouth. Community Dent Health. 1999;16(1):12–7. 10697349

[10] Thomson WM, van der Putten G-J, de Baat C, Ikebe K, Matsuda K, Enoki K, et al. Shortening the xerostomia inventory. Oral Surg Oral Med Oral Pathol Oral Radiol Endod. 2011;112(3):322–7. doi: 10.1016/j.tripleo.2011.03.024 21684773 PMC3154566

[11] van der Putten G-J, Brand HS, Schols JMGA, de Baat C. The diagnostic suitability of a xerostomia questionnaire and the association between xerostomia, hyposalivation and medication use in a group of nursing home residents. Clin Oral Investig. 2011;15(2):185–92. doi: 10.1007/s00784-010-0382-1 20165967 PMC3056013

[12] Westgaard KL, Hynne H, Amdal CD, Young A, Singh PB, Chen X, et al. Oral and ocular late effects in head and neck cancer patients treated with radiotherapy. Sci Rep. 2021;11(1):4026. doi: 10.1038/s41598-021-83635-w 33597629 PMC7889862

[13] Chaffee BW, Halpern-Felsher B, Cheng J. E-cigarette, cannabis and combustible tobacco use: associations with xerostomia among California adolescents. Community Dent Oral Epidemiol. 2023;51(2):180–6. doi: 10.1111/cdoe.12721 34927762 PMC9207149

[14] Wang MTM, Thomson WM, Craig JP. Association between symptoms of xerostomia and dry eye in older people. Cont Lens Anterior Eye. 2020;43(2):99–102. doi: 10.1016/j.clae.2019.09.002 31542280

[15] He S, Wang J, Li M. Validation of the Chinese version of the Summated Xerostomia Inventory (SXI). Qual Life Res. 2013;22(10):2843–7. doi: 10.1007/s11136-013-0420-y 23619629

[16] Lapnimitanun C, Wiriyakijja P, Matangkasombut O, Komin O. Cross-cultural adaptation of the Thai Xerostomia Inventory and Summated Xerostomia Inventory. Oral Dis. 2024;30(7):4331–40. doi: 10.1111/odi.14891 38342965

[17] Hohenberger R, Baumann I, Plinkert PK, Brinster R, Krisam J, Affolter A, et al. Validating the Xerostomia Inventory in a radiation-induced xerostomia population in German language. Oral Dis. 2019;25(7):1744–50. doi: 10.1111/odi.13154 31295368

[18] Moon S, Oh E, Chung D, Choi R, Hong G-RS. Validation of the Korean version of the Summated Xerostomia Inventory among older adults residing in nursing homes. BMC Public Health. 2024;24(1):1466. doi: 10.1186/s12889-024-18875-2 38822313 PMC11143705

[19] Thomson WM, Brown RH, Williams SM. Medication and perception of dry mouth in a population of institutionalised elderly people. N Z Med J. 1993;106(957):219–21. 8367076

[20] Fox PC, Busch KA, Baum BJ. Subjective reports of xerostomia and objective measures of salivary gland performance. J Am Dent Assoc. 1987;115(4):581–4. doi: 10.1016/s0002-8177(87)54012-0 3477595

[21] Närhi TO. Prevalence of subjective feelings of dry mouth in the elderly. J Dent Res. 1994;73(1):20–5. doi: 10.1177/00220345940730010301 8294614

[22] Locker D. Oral health and quality of life. Oral Health Prev Dent. 2004;2 Suppl 1:247–53. 15646581

[23] Population Assessment of Tobacco and Health (PATH) Study [United States] Public-Use Files. Wave 6: Adult Questionnaire (English Version). ICPSR.

[24] Silva MF, Nascimento GG, Leite FRM, Horta BL, Demarco FF. Periodontitis and self-reported halitosis among young adults from the 1982 Pelotas Birth Cohort. Oral Dis. 2020;26(4):843–6. doi: 10.1111/odi.13286 31981254

[25] Carney CE, Buysse DJ, Ancoli-Israel S, Edinger JD, Krystal AD, Lichstein KL. The consensus sleep diary: standardizing prospective sleep self-monitoring. Sleep. 2012;35(2):287–302. 22294820 10.5665/sleep.1642 PMC3250369

[26] Cohen S, Williamson G. Perceived stress in a probability sample of the United States. In: Spacapan S, Oskamp S (Eds) The Social Psychology of Health. Newbury Park, CA: Sage; 1988.

[27] Baker FB, Kim SH. The basics of item response theory using R. Cham: Springer International Publishing; 2017.

[28] Scholtes VA, Terwee CB, Poolman RW. What makes a measurement instrument valid and reliable?. Injury. 2011;42(3):236–40. doi: 10.1016/j.injury.2010.11.042 21145544

[29] Staffini A, Fujita K, Svensson AK, Chung U-I, Svensson T. Statistical methods for item reduction in a representative lifestyle questionnaire: pilot questionnaire study. Interact J Med Res. 2022;11(1):e28692. doi: 10.2196/28692 35302507 PMC8976253

[30] Atif S, Syed SA, Sherazi UR, Rana S. Determining the relationship among stress, xerostomia, salivary flow rate, and the quality of life of undergraduate dental students. J Taibah Univ Med Sci. 2020;16(1):9–15. doi: 10.1016/j.jtumed.2020.10.019 33603626 PMC7858027

[31] Bulthuis MS, Jan Jager DH, Brand HS. Relationship among perceived stress, xerostomia, and salivary flow rate in patients visiting a saliva clinic. Clin Oral Investig. 2018;22(9):3121–7. doi: 10.1007/s00784-018-2393-2 29520470 PMC6224012

[32] Bergdahl M, Bergdahl J. Low unstimulated salivary flow and subjective oral dryness: association with medication, anxiety, depression, and stress. J Dent Res. 2000;79(9):1652–8. doi: 10.1177/00220345000790090301 11023259

[33] Apessos I, Andreadis D, Steiropoulos P, Tortopidis D, Angelis L. Investigation of the relationship between sleep disorders and xerostomia. Clin Oral Investig. 2020;24(5):1709–16. doi: 10.1007/s00784-019-03029-1 31372830

[34] Dawes C. Circadian rhythms in human salivary flow rate and composition. J Physiol. 1972;220(3):529–45. doi: 10.1113/jphysiol.1972.sp009721 5016036 PMC1331668

[35] Hilditch CJ, McEvoy RD, George KE, Thompson CC, Ryan MK, Rischmueller M, et al. Upper airway surface tension but not upper airway collapsibility is elevated in primary Sjögren’s syndrome. Sleep. 2008;31(3):367–74. doi: 10.1093/sleep/31.3.367 18363313 PMC2276736

[36] Santiago PHR, Song Y, Hanna K, Nair R. Degrees of xerostomia? A Rasch analysis of the Xerostomia Inventory. Community Dent Oral Epidemiol. 2020;48(1):63–71. doi: 10.1111/cdoe.12504 31715641

