# Supplementary material for: A brief xerostomia inventory for young adults
Source: PLoS One. 2026 May 20;21(5):e0349869. doi: 10.1371/journal.pone.0349869 (PMC13189318; doi:10.1371/journal.pone.0349869)
Supplement: S1 File — This Supporting Information files contains the additional figures S1 Fig, through S5 Fig. and their captions. (PDF) [file pone.0349869.s001.pdf]

**Supporting Information**
***A Brief Xerostomia Inventory for Young Adults***

S1 Figure. Exploratory Factor Analysis: Scree Plot

S2 Figure. Item Information Functions

S3 Figure. Summed Scale Information Functions

S4 Figure. Young Adult Shortened Xerostomia Inventory Sum Scores and a Global Xerostomia Item

S5 Figure. Receiver Operating Characteristic Curves for Two Shortened Xerostomia Inventories

**Supplemental Figure 1. Exploratory Factor Analysis: Scree Plot**

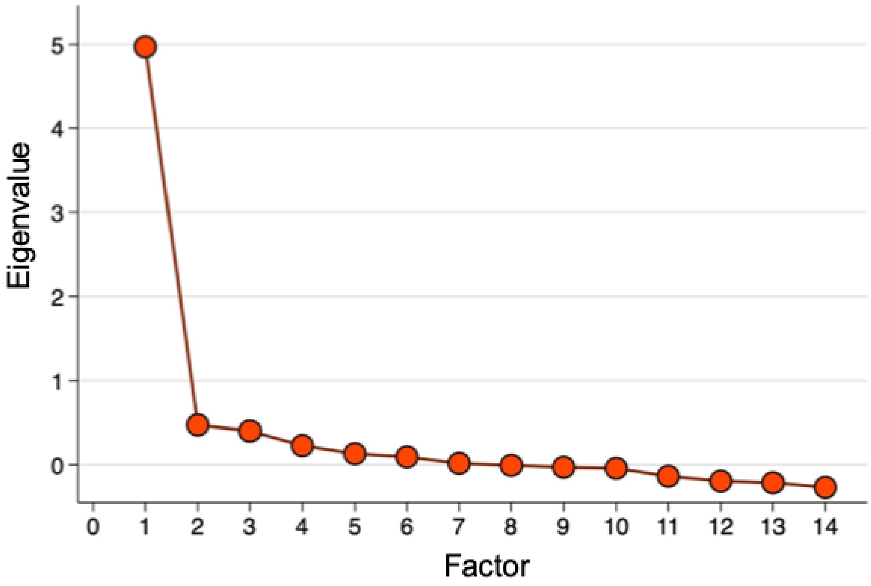


In exploratory factor analysis, one factor had an eigen value of 4.97 and explained 91.8% of the variance. No other factor had an Eigenvalue greater than 0.5, suggesting that a unidimensional model is reasonable for the data. This implies that the xerostomia scale items measure one latent construct (experience of dry mouth).

**Supplemental Figure 2. Item Information Functions**

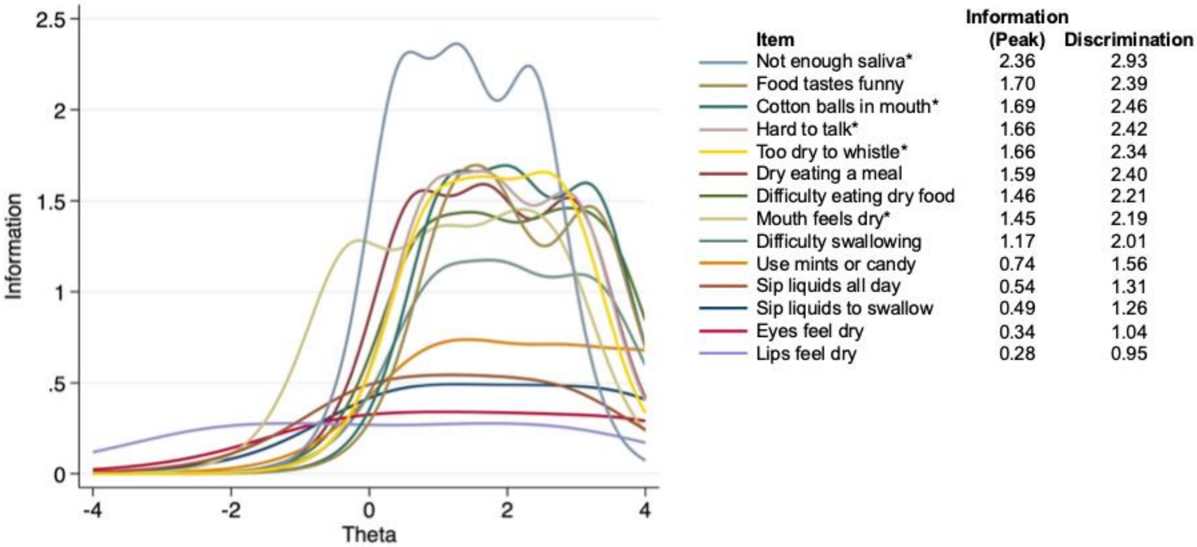


Based on a graded 2-parameter logistic item response theory model, each individual xerostomia item was plotted according to difficulty (theta) and information. Higher discrimination items provided more differentiation between less/more severe xerostomia at a given level of theta.
* = items retained for the Young Adult Shortened Xerostomia Inventory (SXI-Y)

**Supplemental Figure 3. Summed Scale Information Functions**

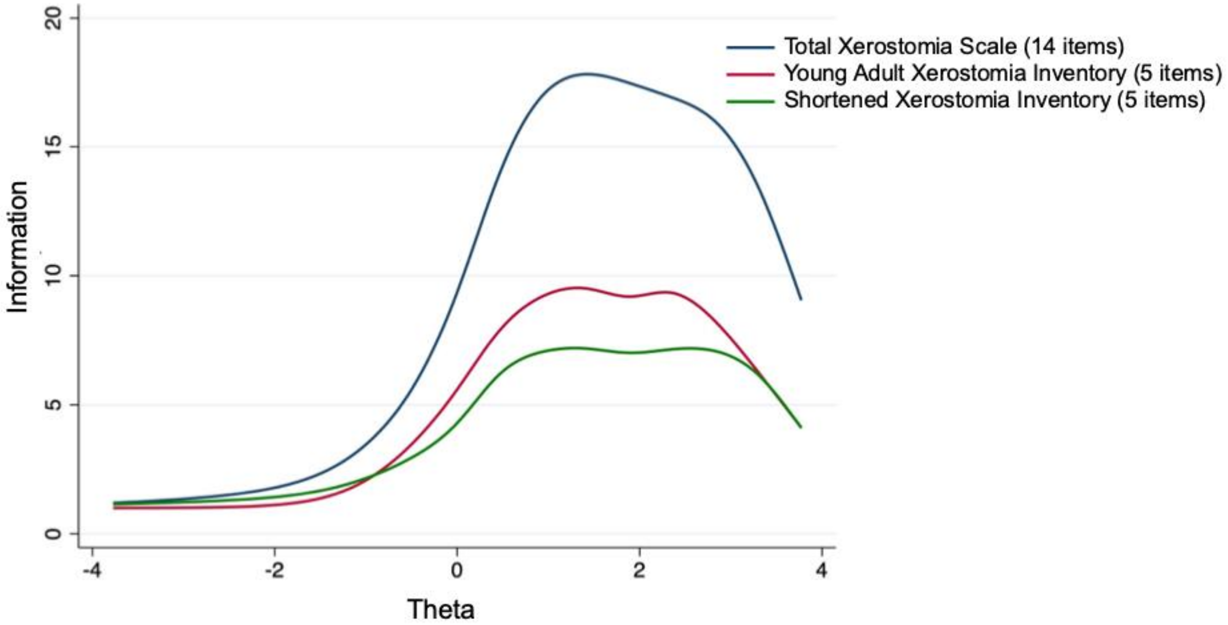


Based on the graded 2-parameter logistic item response theory model, summed xerostomia scales were plotted according to difficulty (theta) and information. Compared to the 5 items included in the original Shortened Xerostomia Inventory (SXI), in this population, the 5 items included in the proposed Young Adult Shortened Xerostomia Inventory (SXI-Y) maintained a larger portion of the information provided by the 14 items in the total scale (at a similar level of theta).

**Supplemental Figure 4. Young Adult Shortened Xerostomia Inventory Sum Scores and a Global Xerostomia Item**

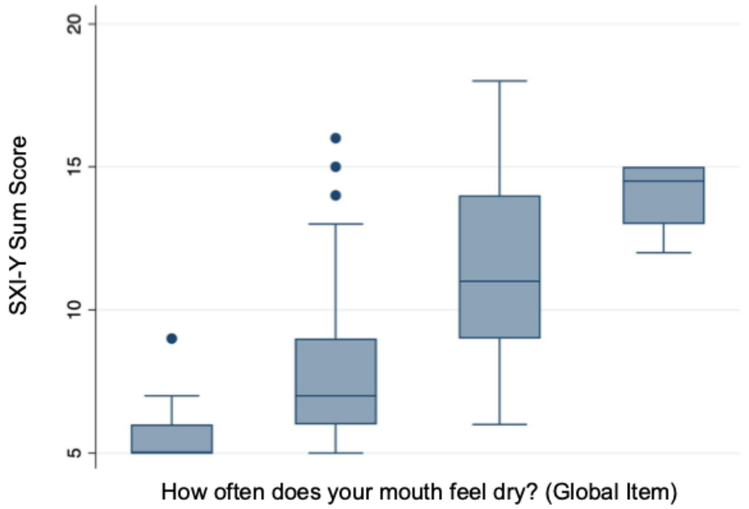


| | Never | Occasionally | Frequently | Always |
|---|---|---|---|---|
| N | 92 | 159 | 23 | 4 |
| SXI-Y (mean) | 5.4 | 7.8 | 11.3 | 14.0 |
| SXI-Y (median) | 5 | 7 | 11 | 14.5 |

Sum scores on the Young Adult Shortened Xerostomia Inventory (SXI-Y) were higher on average, in a stepwise pattern over each level of a single global dry mouth survey item ("How often does your mouth feel dry?").

**Supplemental Figure 5. Receiver Operating Characteristic Curves for Two Shortened Xerostomia Inventories**

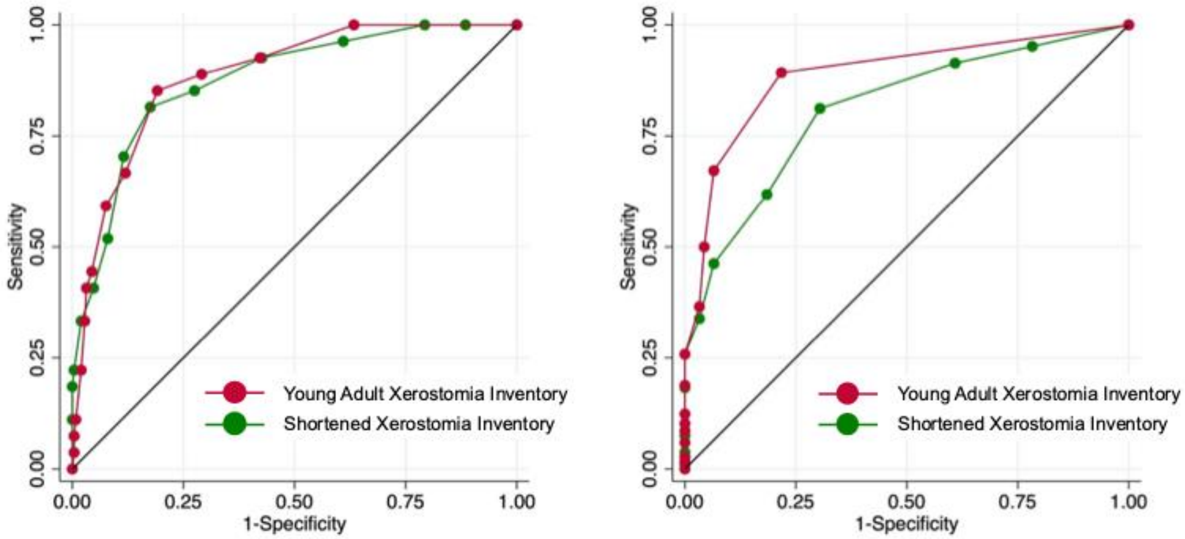


Receiver Operating Characteristic Curves for the proposed Young Adult Shortened Xerostomia Inventory (SXI-Y) and the original Shortened Xerostomia Inventory (SXI) against a gold standard of experiencing mouth dryness frequently or always (LEFT) and against a gold standard of experiencing mouth dryness occasionally, frequently, or always (RIGHT).
